# Supplementary material for: A Functionalized Polysaccharide from Sphingomonas sp. HL-1 for High-Performance Flocculation
Source: Polymers (Basel). 2022 Dec 23;15(1):56. doi: 10.3390/polym15010056 (PMC9853492; doi:10.3390/polym15010056)
Supplement: Supplementary file 1 [file polymers-15-00056-s001.zip › polymers-1998870-supplementary.pdf]

A Functionalized Polysaccharide from *Sphingomonas* sp. HL-1 for High-Performance Flocculation

Haolin Huang [1], Jingsong Li [1], Weiyi Tao [2] and Shuang Li [1,*]

[1] College of Biotechnology and Pharmaceutical Engineering, Nanjing Tech University, Nanjing 211816, China
[2] College of Food Science and Light Industry, Nanjing Tech University, Nanjing 211816, China
* Correspondence: lishuang@njtech.edu.cn; Tel./Fax: +86-25-58139942

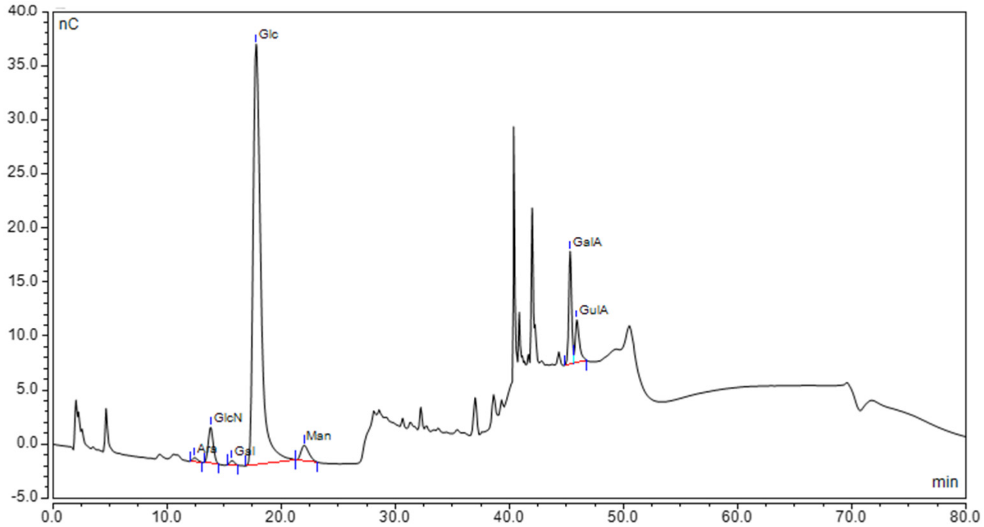

Figure S1. The monosaccharide composition analysis of HL1-PS.
